# Hidden Burden of Lipid Accumulation: A Clinical Case of Niemann-Pick Disease

**DOI:** 10.7759/cureus.95897

**Published:** 2025-11-01

**Authors:** Rafeef A Ghonaimat, Abdullah Shneikat, Bashar M Foudeh, Ibrahim Alsamhouri

**Affiliations:** 1 Department of Internal Medicine, Islamic Hospital, Amman, JOR; 2 Department of Surgery, Islamic Hospital, Amman, JOR

**Keywords:** genetics, hepatosplenomegaly, inherited diseases, internal medicine, lysosomal lipid storage disease, metabolism

## Abstract

We showcase a 29-year-old man who had been previously diagnosed with hypertriglyceridemia. He presented with vague symptoms of generalized weakness, headache, and a feeling of hotness. Investigations revealed the presence of pancytopenia and hepatosplenomegaly, which led to further workup. The cause was identified as a rare, underrecognized diagnosis of Niemann-Pick disease, a lipid storage disorder. This case elucidates the importance of thoroughly looking into signs and symptoms to reach a diagnosis and the need to recognize inherited metabolic disorders as part of unique clinical presentations, which will lead to overall better patient care and outcomes.

## Introduction

Lipid storage disorders are varied entities connected by their molecular pathophysiology. Each disorder is caused by a hereditary lysosomal hydrolase deficiency that results in the lysosomal buildup of the particular enzyme's sphingolipid substrate in various organs [1,2].

The pathways of glycosphingolipid metabolism in the visceral organs and nervous tissue are clarified, and a genetically related metabolic derangement is identified for each catabolic phase [3].

Examples of lipid storage disorders include GM1 gangliosidoses [4], GM2 gangliosidoses [4], Gaucher disease, sphingomyelinase deficiency or Niemann-Pick disease (NPD) types A and B [4], NPD type C, Fabry disease, fucosidosis, Schindler disease, and others.

Sphingomyelin-cholesterol lipidosis, commonly referred to as NPD, is a collection of autosomal recessive disorders characterized by widespread clinical presentations including splenomegaly, varying neurologic impairments, and the storage of lipids, particularly cholesterol and sphingomyelin [1].

NPD type A is the acute neuronopathic variant, while NPD type B is less severe and has a delayed onset. Ashkenazi Jews have the highest incidence of NPD type A; their estimated gene frequency is 1:100. It is estimated that the combined prevalence of types A and B is 1:250,000 [5].

Due to the rarity of and wide spectrum of clinical presentations of lipid storage disorders, the need to identify and report various approaches to establishing diagnoses, especially at facilities where specific enzyme assays are unavailable, is now of great importance.

## Case presentation

A 29-year-old man of Middle Eastern origin who had been previously diagnosed with hypertriglyceridemia (off treatment) but no other medical illnesses, with no family history of any chronic or inherited medical illnesses, presented complaining of a headache of one week prior to admission, which was dull, band-like, gradually progressive, and relieved by the use of simple analgesia. In addition, the patient admitted to having intermittent feelings of hotness, which were not measured, associated with excessive night sweats. He also reported having generalized body weakness that involved his entire body, which was gradual, progressive, and affecting his daily activities and required him to seek medical attention at our hospital.

Upon clinical assessment, the patient exhibited full consciousness, alertness, and orientation. His vital signs were within acceptable limits as follows: temperature, 36.6°C orally; blood pressure, 130/75 mmHg; heart rate, 71 beats per minute and regular; respiratory rate, 19 breaths per minute; and oxygen saturation, 98% on room air. He weighed 92 kg and his BMI was 28. Pallor was present but no jaundice. Hepatomegaly (regular edge, smooth surface, and nontender) and splenomegaly that extended to the umbilicus were seen. Furthermore, the patient had no chest deformities and had symmetrical chest expansion with resonant percussion and good bilateral air entry; his heart sounds were regular S1 and S2 with no murmurs. On neurologic examination, he had intact power and sensation in all limbs, displayed intact cranial nerves, and had negative cerebellar signs. The patient had no palpable lymphadenopathy.

On day 1, the patient underwent biochemical analysis as shown in Table 1, highlighting pancytopenia with a decreased total leukocyte count of 1.9×10^9^/L with 30% lymphocytes and 56% neutrophils, compared to an adult's typical reference range of 4-11×10^9^/L. There were 77×10^9^/L platelets (normal range: 150-450×10^9^/L). The hemoglobin level was 12.6 g/dL (normal range: 13.2-16.6 g/dL).

**Table 1:** Initial laboratory results HB: hemoglobin; WBC: white blood cells; CRP: C-reactive protein; HDL: high-density lipoprotein; LDL: low-density lipoprotein

Laboratory test	Result	Reference range
Platelet count	77×10^9^/L	150-450×10^9^/L
HB	12.6 g/dL	13.2-16.6 g/dL (male)
WBC	1.9×10^9^/L	4-11×10^9^/L
Neutrophils	56%	40-75%
Lymphocytes	30%	20-45%
Urea	36	Normal
Creatinine	1.4 mg/dL	0.6-1.1 mg/dL
Sodium	138 mEq/L	135-145 mEq/L
Potassium	4.1 mEq/L	3.7-5.2 mEq/L
Chloride	101 mEq/L	96-106 mEq/L
CRP	22 mg/L	<10 mg/L
Triglycerides	331 mg/dL	<150 mg/dL
Cholesterol, total	104 mg/dL	<200 mg/dL
Cholesterol, HDL	13 mg/dL	>40 mg/dL
Cholesterol, LDL	38 mg/dL	<100 mg/dL
Non-HDL cholesterol	91 mg/dL	<130 mg/dL
Total protein	7 g/dL	6.4-8.3 g/dL
Albumin	5.11 g/dL	3.5-5.2 g/dL
Total bilirubin	2.21 mg/dL	Up to 1 mg/dL
Direct bilirubin	0.57 mg/dL	Up to 0.3 mg/dL
Alkaline phosphatase	60 u/L	35-104 u/L
Alanine transaminase	18 u/L	Up to 33 u/L
Aspartate transaminase	28 u/L	Up to 32 u/L
Gamma-glutamyl transferase	18 u/L	<40 u/L

On day 2, repeat blood tests were requested as shown in Table 2, highlighting a slight improvement in his total leukocyte and platelet count in addition to decreasing bilirubin levels. Furthermore, additional tests were ordered as mentioned in Table 3 to further evaluate the presence of hepatosplenomegaly, which carries a wide differential diagnosis, including but not limited to infection, malignancy, and multisystem disorders.

**Table 2:** Follow-up laboratory results HB: hemoglobin; WBC: white blood cells

Laboratory test	Result	Reference range
Platelet count	101×10^9^/L	150-450×10^9^/L
HB	12.6 g/dL	13.2-16.6 g/dL (male)
WBC	2.8×10^9^/L	4-11×10^9^/L
Neutrophils	49%	40-75%
Lymphocytes	41%	20-45%
Total bilirubin	0.86 mg/dL	Up to 1 mg/dL
Direct bilirubin	0.29 mg/dL	Up to 0.3 mg/dL

**Table 3:** Further laboratory tests PT: prothrombin time; INR: international normalized ratio; PTT: partial thromboplastin time; HBsAg: hepatitis B surface antigen; anti-HCV: anti-hepatitis C virus; HIV: human immunodeficiency virus; LDH: lactate dehydrogenase; 2ME: 2-mercaptoethanol

Laboratory test	Result	Reference range
PT	17.2 seconds	11-13.5 seconds
INR	1.32	0.8-1.2
PTT	33 seconds	25-35 seconds
Hepatitis B (HBsAg)	Negative	Negative
Hepatitis C (anti-HCV)	Negative	Negative
HIV	Negative	Negative
LDH	274 U/L	140-280 U/L
*Brucella melitensis *(total)	Negative	-
*Brucella melitensis* (2ME)	Negative	-
*Brucella abortus* (total)	Negative	-
*Brucella abortus* (2ME)	Negative	-

**Figure 1:**
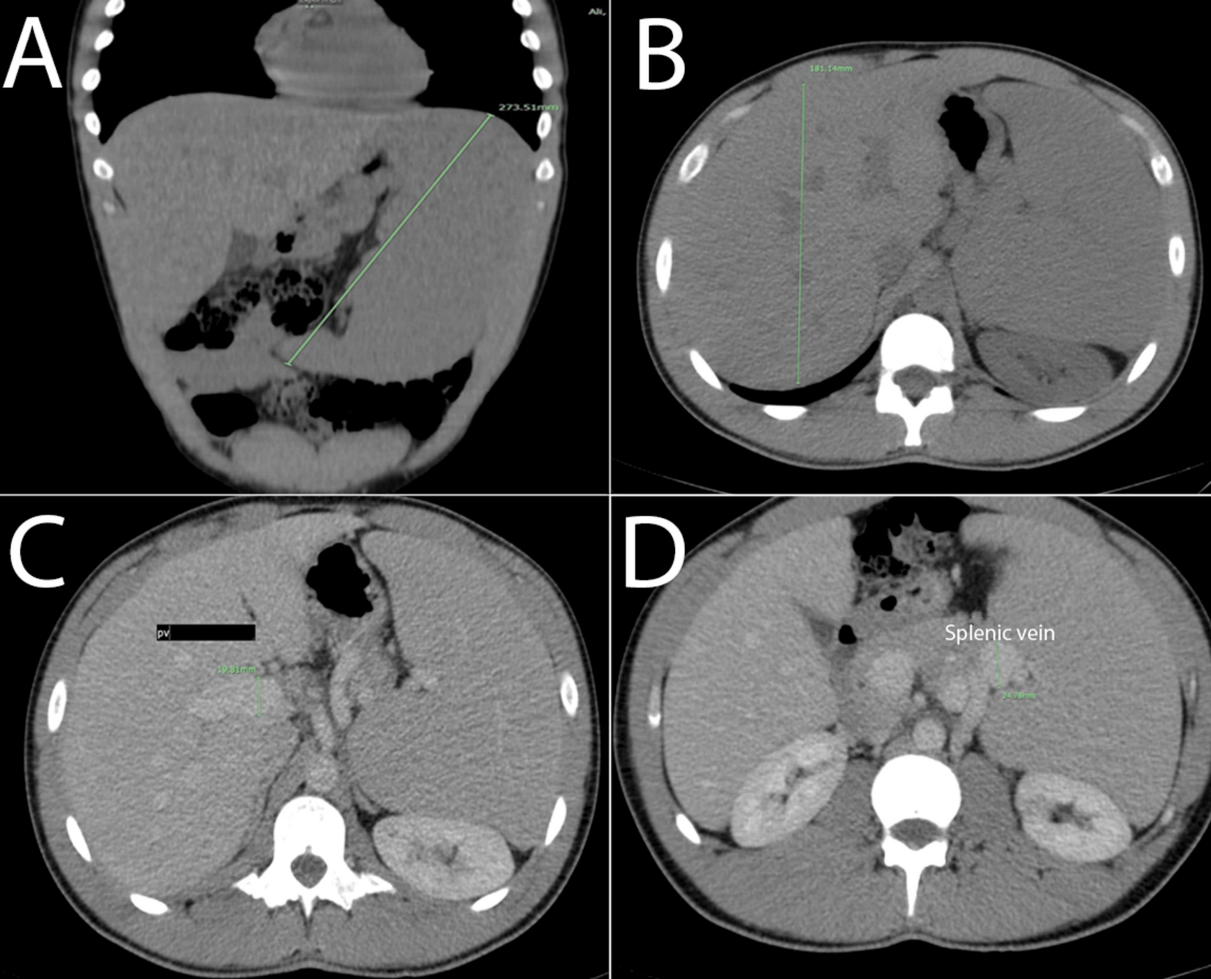
Abdominal CT scan Abdominal CT scan reveals splenomegaly measuring 27 cm craniocaudally (A), hepatomegaly measuring 18.5 cm in anteroposterior diameter (B), dilatation of the portal vein and its confluent measuring 2 cm in anteroposterior diameter (C), and dilatation of the splenic vein reaching 2.5 cm in anteroposterior diameter (D)

According to the supervising team's clinical impression, imaging was required. The patient had undergone an abdominal ultrasound at another facility, which showed the presence of hepatosplenomegaly, so further evaluation was needed. An abdominal CT with IV contrast (Figure 1) was requested and showed an enlarged spleen measuring 27 cm along with a small, well-defined hypodense lesion measuring 0.9×0.8 cm in the lateral aspect of the middle part. Also, the liver was enlarged in size, measuring 18.5 cm. Additionally, dilatation of the portal vein and its confluent, measuring 2 cm, and the splenic vein, reaching 2.5 cm, in anteroposterior diameter were seen. 

**Figure 2:**
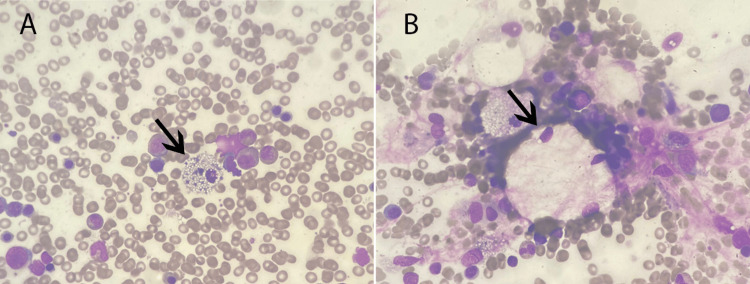
Bone marrow aspirate (A-B) Bone marrow aspirate, Wright's stain: The smears reveal the following: many particles of normocellular bone marrow, erythroid hyperplasia with normal maturation, active granulopoiesis with normal maturation, megakaryocytes normal in morphology, no increase of blasts, lymphocytes, or plasma cells, and few foamy large macrophages (arrows)

**Figure 3:**
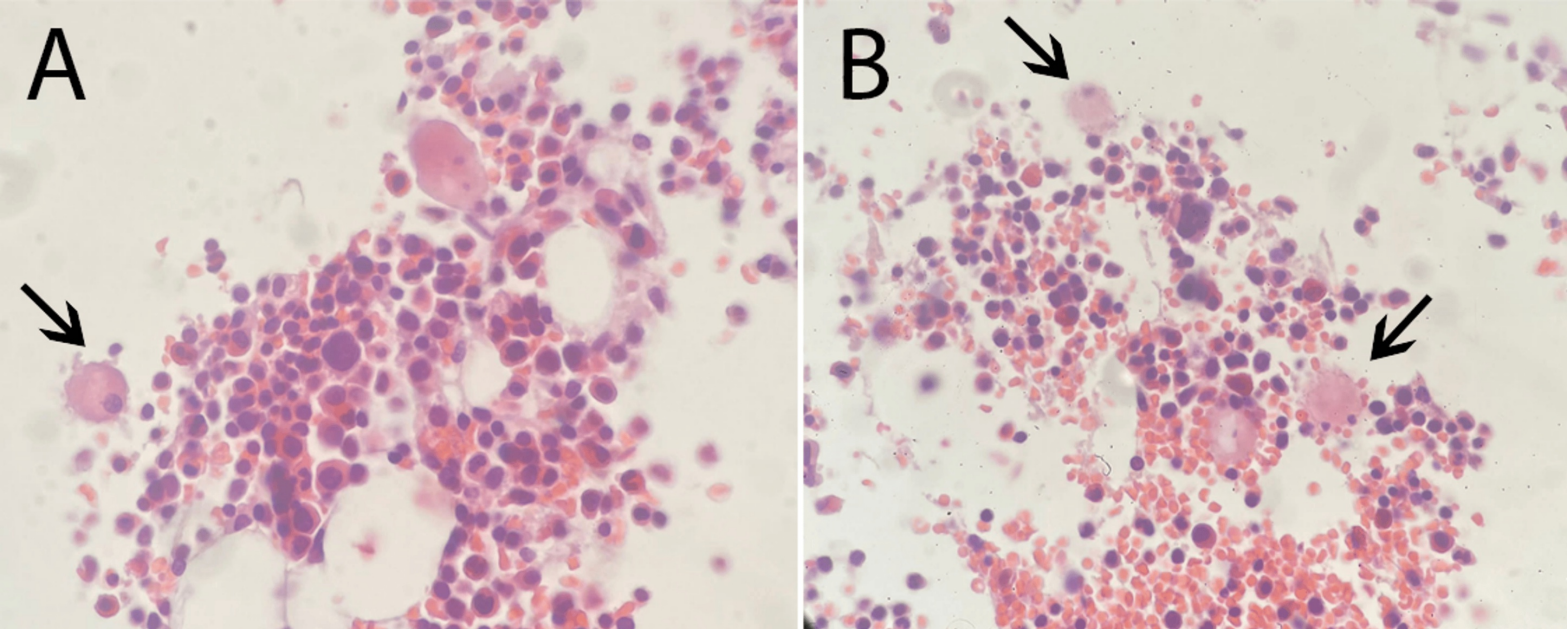
Bone marrow trephine biopsy (A-B) Bone marrow trephine biopsy, H&E stain: Adequate sample. Sections reveal a normocellular bone marrow (60% cellularity) with normal trilineage hematopoiesis. Megakaryocytes are normal in number and morphology. A small population of foamy macrophages is seen (arrows), suggestive of early marrow involvement by Niemann-Pick disease of sea-blue histiocytosis. No marrow fibrosis is seen

The above findings imposed a great need for the supervising team to rule out malignancy and infiltrative diseases of the bone marrow. A bone marrow biopsy was done (Figures 2-3). The findings are described and shown below.

The patient's clinical condition had improved after receiving symptomatic treatment over the course of his stay at the hospital. After receiving the presumed diagnosis of NPD type B, it was explained to the patient that he probably has a rare genetic disorder, which would need further testing with an enzyme assay to confirm, which was not available at our facility. Hence, the patient was then advised to continue follow-up as an outpatient to monitor recurring symptoms and disease progression, received counseling on possible complications related to this disease, and was discharged home. 

## Discussion

According to its histology, NPD was classified as reticuloendotheliosis but is currently categorized into two main subtypes. The pathogenic mutations in the sphingomyelin phosphodiesterase-1 (SMPD1) gene cause NPD types A and B, which are allelic disorders that involve a primary reduction of acid sphingomyelinase activity. The severe, early-onset variant is known as NPD type A, while the less severe, later-onset variant is known as NPD type B [6]. Additionally, a phenotype that falls in between NPD types A and B has been identified [7,8]. Acid sphingomyelinase deficiency (ASMD) has been proposed to include the NPD type A and B disease subtypes [6].

NPD type A is the acute neuronopathic form. The incidence of NPD type A is highest among Ashkenazi Jews, in whom the gene frequency is estimated to be 1:100. The overall prevalence of ASMD (types A and B combined) is estimated to be 1:250,000 [9]. In NPD type A, affected patients have feeding difficulties, early motor skill deficits, and hepatosplenomegaly. By the time an affected individual is two or three years old, there is a significant, rapid, and progressive deterioration of neurologic function, which ultimately results in death.

Patients with NPD type B tend to reach adulthood and have better chances of survival compared with NPD type A patients [7]. It has a characteristic of developing hepatosplenomegaly during infancy or childhood. Thrombocytopenia as a result of hypersplenism is also a manifestation. Infiltration of foamy histiocytes, ballooning of hepatocytes, and fibrosis of the liver result in severe hepatic manifestations [9]. Shortened height with delayed skeletal maturation, interstitial lung disease, hyperlipidemia, and ocular abnormalities such as macular halos and cherry red maculae are examples of other system manifestations [10-12]. Progressive hypersplenism and gradual deterioration of pulmonary function take time but will eventually develop [13,14].

The diagnosis of NPD is challenging, and it could be suggested by careful examination and search of possible clinical features, including but not limited to hepatosplenomegaly and interstitial lung disease [6]. NPD type A has a wide spectrum of possible presentations, including early developmental delay, while hyperlipidemia and thrombocytopenia could suggest NPD type B [6].

In addition to taking the history into account and the use of molecular genetic testing to identify both disease-causing alleles in the SMPD1 gene, another method to confirm diagnosis includes the measurement of residual acid sphingomyelinase activity in peripheral blood leukocytes or cultured skin fibroblasts, which correlates to less than 10% of controls [6,15]. In situations in which limited resources are available and neither of those methods is available for diagnosis, clinical scenarios that suggest the possible diagnosis of lipid storage disease warrant the use of tissue biopsy, which was utilized in this case, with bone marrow results showing evidence of foamy macrophages.

## Conclusions

This case represented a male patient who complained of non-specific symptoms and who had findings of pallor and hepatosplenomegaly on examination. Further testing using available techniques revealed the presence of pancytopenia and foamy macrophages. The constellation of non-specific signs and symptoms coupled with unique bone marrow findings urged the need to formulate a specific differential diagnosis. However, physicians must bear in mind the limited availability of resources at certain facilities and the importance of navigating a wide range of both common and rare disorders. This also served as a reminder to include various metabolic disorders when dealing with patients presenting with a constellation of non-specific signs and symptoms. 
